# How to risk-stratify elective surgery during the COVID-19 pandemic?

**DOI:** 10.1186/s13037-020-00235-9

**Published:** 2020-03-31

**Authors:** Philip F. Stahel

**Affiliations:** Department of Specialty Medicine, Rocky Vista University, College of Osteopathic Medicine, Parker, CO 80134 USA

**Keywords:** Coronavirus, COVID-19, Resource utilization, Emergency preparedness, Elective surgery

On March 11, 2020, the World Health Organization (WHO) declared the novel coronavirus disease 2019 (COVID-19) a global pandemic, which classifies the outbreak as an international emergency [1]. At the time of drafting this editorial, COVID-19 has swept through more than 115 countries and infected over 200,000 people around the globe [2–4]. More than 7000 individuals have died during the early phase of the pandemic, implying a high estimated case-fatality rate of 3.5% [2–4]. The rapidly spreading outbreak imposes an unprecedented burden on the effectiveness and sustainability of our healthcare system. Acute challenges include the exponential increase in emergency department (ED) visits and inpatient admission volumes, in conjunction with the impending risk of health care workforce shortage due to viral exposure, respiratory illness, and logistical issues due to the widespread closure of school systems [5]. Subsequent to the WHO declaration, the United States Surgeon General proclaimed a formal advisory to cancel elective surgeries at hospitals due to the concern that elective procedures may contribute to the spreading of the coronavirus within facilities and use up medical resources needed to manage a potential surge of coronavirus cases [6]. The announcement escalated to a nationwide debate regarding the safety and feasibility of continuing to perform elective surgical procedures during the COVID-19 pandemic [7, 8]. Many health care professionals erroneously interpreted the Surgeon General’s recommendation as a “blanket directive” to cancel all elective procedures in the Country [9]. This notion was vehemently challenged in an open letter to the Surgeon General on behalf of United States hospitals [10]. The letter outlined a significant concern that the recommendation could be “interpreted as recommending that hospitals immediately stop performing elective surgeries without clear agreement on how we classify various levels of necessary care “[10]. Notably, the Surgeon General’s recommendation was based on a preceding statement by the American College of Surgeons (ACS) with a call to prioritize appropriate resource allocation during the coronavirus pandemic as it relates to elective invasive procedures.

The ACS bulletin stated the following specific recommendations [11]:

- *Each hospital, health system, and surgeon should thoughtfully review all scheduled elective procedures with a plan to minimize, postpone, or cancel electively scheduled operations, endoscopies, or other invasive procedures until we have passed the predicted inflection point in the exposure graph and can be confident that our health care infrastructure can support a potentially rapid and overwhelming uptick in critical patient care needs.*
  - *Immediately minimize use of essential items needed to care for patients, including but not limited to, ICU beds, personal protective equipment, terminal cleaning supplies, and ventilators. There are many asymptomatic patients who are, nevertheless, shedding virus and are unwittingly exposing other inpatients, outpatients, and health care providers to the risk of contracting COVID-19.*

Importantly, the notion to “thoughtfully review all scheduled elective procedures “does not reflect on a presumed imperative to cancel all elective surgical cases across the United States [11]. The uncertainty on the predicted time course of COVID-19 beyond a critical inflection point implies that patients may be deprived of access to timely surgical care likely for many months to come. Arguably, the potential fallout from inconsiderate elective surgery cancellations may have a more dramatic and immeasurable impact on the health of our communities than the morbidity and mortality inflicted by the novel coronavirus disease. For the sake of this discussion, it is imperative to understand that the term “elective “surgery does not mean optional surgery, and rather implies that a procedure is not immediately indicated in response to a limb- or life-threatening emergency. A current estimate suggests that more than 50% of all elective surgical cases have a potential to inflict significant harm on patients if cancelled or delayed [12]. The physiological condition of a vulnerable cohort of patients may rapidly worsen in absence of appropriate surgical care, and the resulting decline in patients‘health will likely make them more vulnerable to a coronavirus infection [12].

A recent publication from the Naval Medical University in Shanghai reported on the inherent risks of delaying surgery for colorectal cancer during the COVID-19 outbreak in China [13]. In addition, impressive anecdotal reports of individual patient stories illustrate the unintended consequences imposed by cancelling scheduled surgery, as exemplified by a woman who stated that she felt like there was a “time bomb” inside her after surgery for early stage cervical cancer had been cancelled and indefinitely postponed [14]. Unequivocally, many elective non-urgent surgeries will become urgent at some point in time, depending on how long the COVID-19 outbreak will prevail. Dr. David Hoyt, a trauma surgeon and executive director of the ACS, recently stated:” Right now, most people are planning for a time period of 4–6 weeks for the peak to hit, but nobody really knows. We’re using our best judgment on the fly.” [11].

In light of all the underlying assumptions and uncertainties, it appears imperative to design and implement clinically relevant and patient safety-driven algorithms to guide the decision-making for appropriate surgical care. Elective procedures can pragmatically be stratified into “essential“, which implies that there is an increased risk of adverse outcomes by delaying surgical care for an undetermined period of time, versus “non-essential “or “discretionary“, which alludes to purely elective procedures that are not time-sensitive for medical reasons. Table 1 provides a suggested stratification by urgency of surgical indications for considering appropriate elective case cancellation. Equivocal surgical cases – which do not fall into either “essential “or “non-essential “categories – appear to have shown an effective self-regulating mechanism in the early phase of the COVID-19 outbreak, driven by patients voluntarily cancelling their scheduled elective procedures and surgeons evaluating appropriate indications on a case-by-case basis [15].

**Table 1:** Examples of surgical case types stratified by indication and urgency

Indication	Urgency	Case examples
Emergent	< 1 h	• Life-threating emergencies • Acute exsanguination / hemorrhagic shock • Trauma level 1 activations • Acute vascular injury or occlusion • Aortic dissection • Emergency C-section • Acute compartment syndrome • Necrotizing fasciitis • Peritonitis • Bowel obstruction / perforation
Urgent	< 24 h	• Appendicitis / cholecystitis • Septic arthritis • Open fractures • Bleeding pelvic fractures • Femur shaft fractures & hip fractures • Acute nerve injuries / spinal cord injuries • Surgical infections
Urgent-elective	< 2 weeks	• Cardiothoracic / cardiovascular procedures • Cerebral aneurysm repair • Vascular access devices • Skin grafts / flaps / wound closures • Scheduled C-section • Closed fractures • Spinal fractures & acetabular fractures
Elective (essential)	1–3 months	• Cancer surgery & biopsies • Subacute cardiac valve procedures • Hernia repair • Hysterectomy • Reconstructive surgery
Elective (discretionary)	> 3 months	• Cosmetic surgery • Bariatric surgery • Joint replacement • Sports surgery • Vasectomy / tubal ligation • Infertility procedures

In essence, during the current time of widespread anxiety around the COVID-19 pandemic [16], a pragmatic guide based on underlying risk stratification and resource utilization will help support our ethical duty of assuring access to timely and appropriate surgical care to our patients, while maintaining an unwavering stewardship for scarce resources and emergency preparedness. Figure 1 provides a tentative decision-making algorithm based on elective surgical indications and predicted perioperative utilization of critical resources, including the consideration for intra−/postoperative blood product transfusions, estimated postoperative hospital length of stay, and the expected requirement for prolonged ventilation and need for postoperative ICU admission.

**Fig. 1:**
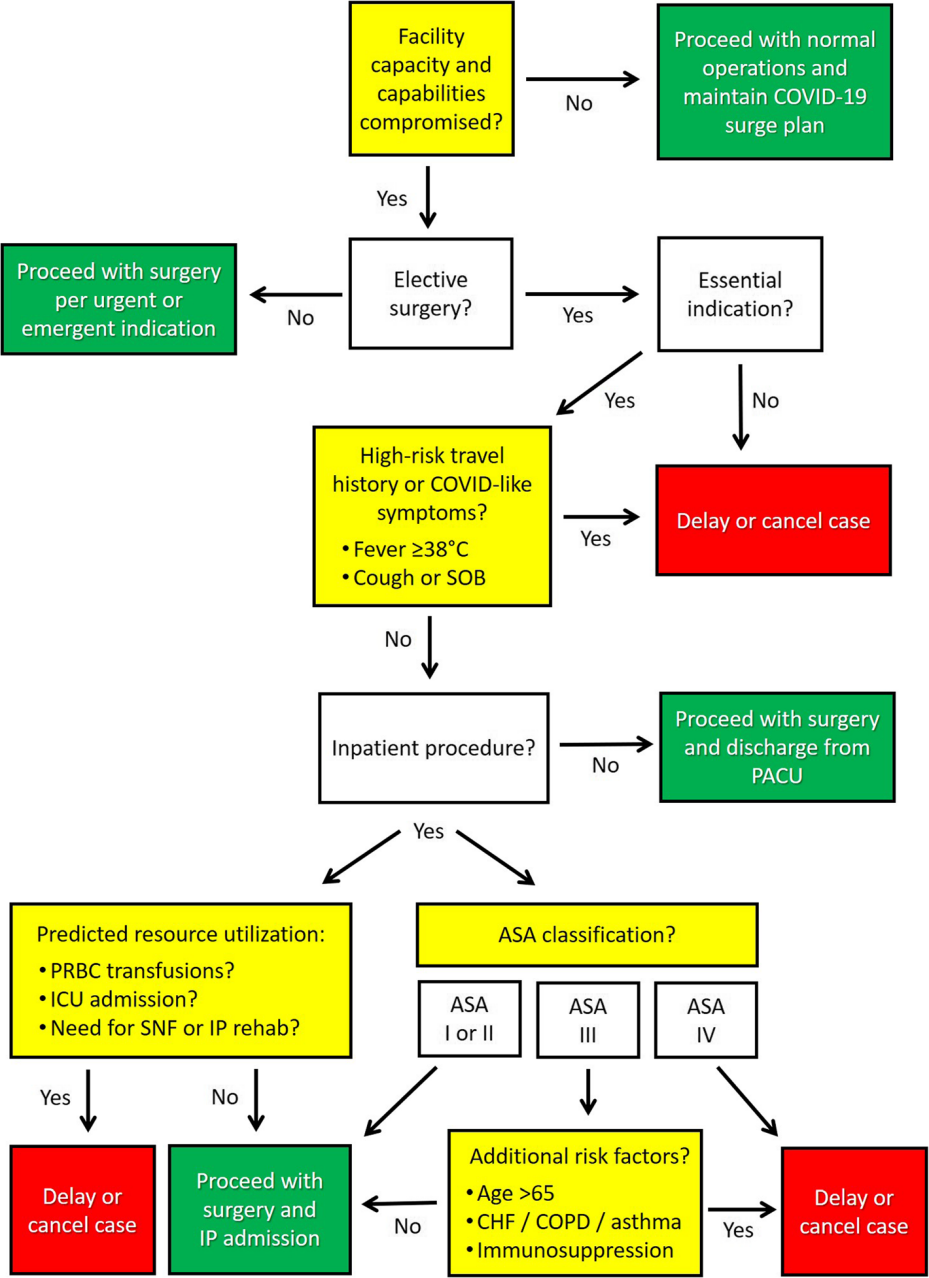
Proposed decision-making algorithm for risk-stratification of elective surgical procedures based on the underlying surgical indication and predicted resource utilization during the current COVID-19 pandemic. Abbreviations: ASA, American Society of Anesthesiologists; CHF, chronic heart failure; COPD, chronic obstructive pulmonary disease; COVID, corona virus disease; ICU, intensive care unit; IP, inpatient; PACU, post-anesthesia care unit; PRBC, packed red blood cells; SNF, skilled nursing facility; SOB, shortness of breath

Ultimately, if rationing of healthcare resources in terms of limiting access to surgical care in the United States will never be needed, then these ongoing crucial discussions will have served as an important exercise in nationwide disaster preparedness.

## Data Availability

Please contact the author for data requests.
